# A Pharmacogenetics Study in Mozambican Patients Treated with Nevirapine: Full Resequencing of *TRAF3IP2* Gene Shows a Novel Association with SJS/TEN Susceptibility

**DOI:** 10.3390/ijms16035830

**Published:** 2015-03-12

**Authors:** Cinzia Ciccacci, Sara Rufini, Sandro Mancinelli, Ersilia Buonomo, Emiliano Giardina, Paola Scarcella, Maria C. Marazzi, Giuseppe Novelli, Leonardo Palombi, Paola Borgiani

**Affiliations:** 1 Department of Biomedicine and Prevention, Genetics Section, University of Rome “Tor Vergata”, Rome 00133, Italy; E-Mails: cinziaciccacci@libero.it (C.C.); sara.rufini@hotmail.it (S.R.); emiliano.giardina@uniroma2.it (E.G.); novelli@med.uniroma2.it (G.N.); 2 Department of Biomedicine and Prevention, Epidemiology Section, University of Rome “Tor Vergata”, Rome 00133, Italy; E-Mails: sandro.mancinelli@uniroma2.it (S.M.); ersilia.buonomo@uniroma2.it (E.B.); paola.scarcella@uniroma2.it (P.S.); leonardo.palombi@gmail.com (L.P.); 3 Laboratory of Molecular Genetics UILDM, Fondazione Santa Lucia, Rome 00179, Italy; 4 Department of Human Sciences, LUMSA University, Rome 00193, Italy; E-Mail: marazzi@lumsa.it

**Keywords:** pharmacogenetics, polymorphisms, Steven–Johnson Syndrome (SJS), Toxic Epidermal Necrolysis (TEN), *TRAF3IP2* gene

## Abstract

Steven–Johnson Syndrome (SJS) and Toxic Epidermal Necrolysis (TEN) are severe adverse drug reactions, characterized by extensive epidermal detachment and erosions of mucous membrane. SJS/TEN is one of the most serious adverse reactions to Nevirapine (NVP) treatment, commonly used in developing countries as first-line treatment of human immunodeficiency virus infection. In the last years *TRAF3IP2* gene variants had been described as associated with susceptibility to several diseases such as psoriasis and psoriatic arthritis. We hypothesized that this gene, involved in immune response and in NF-κB activation, could also be implicated in the SJS/TEN susceptibility. We performed a full resequencing of *TRAF3IP2* gene in a population of patients treated with NVP. Twenty-seven patients with NVP-induced SJS/TEN and 78 controls, all from Mozambique, were enrolled. We identified eight exonic and three intronic already described variants. The case/control association analysis highlighted an association between the rs76228616 SNP in exon 2 and the SJS/TEN susceptibility. In particular, the variant allele (C) resulted significantly associated with a higher risk to develop SJS/TEN (*p* = 0.012 and OR = 3.65 (95% CI 1.33–10.01)). A multivariate analysis by logistic regression confirmed its significant contribution (*p* = 0.027, OR = 4.39 (95% CI 1.19–16.23)). In conclusion, our study suggests that a variant in *TRAF3IP2* gene could be involved in susceptibility to SJS/TEN.

## 1. Introduction

Steven–Johnson Syndrome (SJS) and Toxic Epidermal Necrolysis (TEN) are severe adverse drug reactions, characterized by extensive epidermal detachment and erosions of mucous membrane [1,2,3]. Although these diseases are rare, their incidence is higher in people with human immunodeficiency virus (HIV) infection [4]. These patients have an additional risk also given by the use of Nevirapine (NVP) [5], a non-nucleoside reverse transcriptase inhibitor drug, which is one of the drug most often associated with SJS/TEN development. NVP is commonly used in developing countries as first-line treatment of HIV infection because of its low costs, but unfortunately its use is associated with several adverse reactions such as SJS/TEN, cutaneous rash and liver toxicity [6].

Genetic factors predisposing to SJS/TEN have been identified particularly in the *HLA* (Human Leukocyte Antigen) genes [7,8,9,10]. In our previous studies, we had described associations between SJS/TEN susceptibility and polymorphisms in *CYP2B6* (rs3745274 and rs28399499) and in *HCP5* genes (rs3099844) [11,12].

In recent years, *TRAF3IP2* gene emerged as a critical player for immune responses to pathogens, inflammatory signals and autoimmunity in mammals [13,14,15,16]. The gene codes for a protein involved in the activation of NF-κB and JNK signaling. GWAs studies described this gene as associated with psoriasis and psoriatic arthritis susceptibility [17,18,19]. In previous studies, we had investigated common polymorphisms in *TRAF3IP2* gene and we found associations with a major risk to develop cutaneous extraintestinal manifestations in inflammatory bowel diseases [20] and with systemic lupus erythematosus [21]. In the present paper, we have performed an association study to assess whether *TRAF3IP2* variants could also contribute to the susceptibility to Nevirapine-induced SJS/TEN in a Mozambican population.

## 2. Results

We analyzed 27 patients that developed SJS/TEN as adverse drug reaction and 78 matched controls. Clinical baseline characteristics of all patients are reported in Table 1.

**Table 1:** Clinical baseline characteristics of patients.

Clinical Characteristics at Baseline	SJS	Controls	All	*p* (SJS/TEN *vs.* Controls)
Age, years	31 (27–36)	32 (27–36)	32 (27–36)	0.8
Viral load, log copies/mL	4.1 (3.2–4.6)	3.58 (2.39–4.43)	3.72 (2.98–4.49)	0.41
CD4 cell count, median cells/mm^3^	467 (326–721)	393 (227–591)	409 (230–634)	0.21
BMI	22.8 (21.5–25.4)	22.8 (21.6–26.3)	22.8 (21.6–25.9)	0.78
ALT baseline U/L	8.6 (6.9–12.8)	10.7 (7.3–15.4)	10.2 (7.1–15.2)	0.28
AST baseline U/L	20.5 (15.9–23.5)	20.3 (16.9–23.6)	20.4 (16.8–23.6)	0.91

Values are reported as median and interquartile range.

By full sequencing of *TRAF3IP2* gene, we identified eight exonic and three intronic variants (see Table 2). All SNPs were already known and all genotypes distributions resulted in Hardy–Weinberg equilibrium. We did not find any novel variations. The case/control association analysis highlighted a significant association between the rs76228616 SNP in exon 2 and the SJS/TEN. In particular, the variant allele (C) was more present in SJS/TEN patients than in controls, resulting significantly associated with a higher risk to develop SJS/TEN both at genotypic and allelic level (OR = 3.6 (95% CI = 1.2–10.95) *p* = 0.027, OR = 3.65 (95% CI = 1.33–10.01) and *p* = 0.012). Another SNP, the rs33980500, showed an OR = 2.2 but the association did not reach the statistical significance (*p* = 0.12).

**Figure 1:**
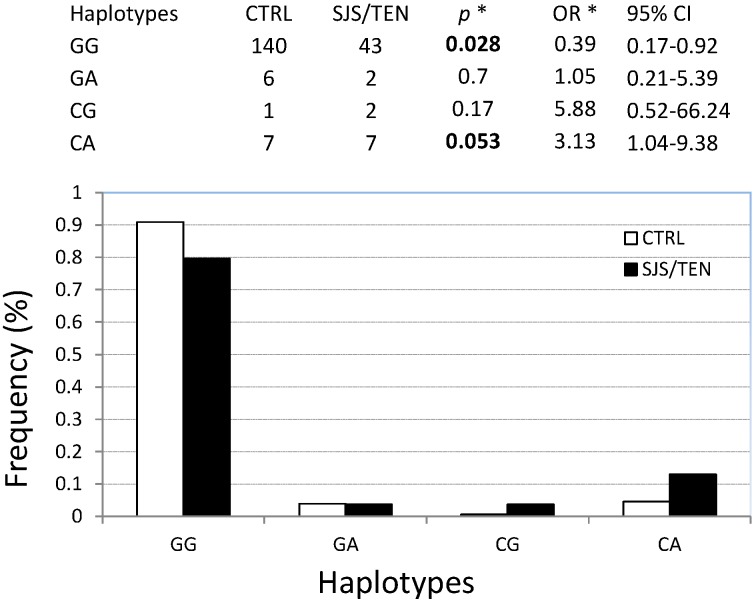
Rs76228616 (G>C)–rs33980500 (G>A) haplotypes distribution in cases and controls. * Each haplotype has been compared *vs.* the sum of the others. Significant *p* values are reported in bold.

In order to elucidate a potential contribution of rs33980500, we inferred the haplotypes between the rs76228616 and rs33980500 and then we compared the haplotypes distribution in controls and cases (Figure 1). The wild type haplotype was significantly associated with a minor risk to develop the disease (OR = 0.39 and *p* = 0.028), whereas the CA haplotypes (constituted by both risk variant alleles) conferred a higher risk to develop the disease (OR = 3.13 and *p* = 0.053). The risk associated with this haplotype is comparable with the risk of the rs76228616 alone; therefore, we concluded that rs33980500 contribution is negligible. The two SNPs are in moderate linkage disequilibrium (D’ = 0.85).

We performed a multivariate analysis by logistic regression considering as dependent variable the presence/absence of SJS/TEN. As independent variables, we included, in addition to the *TRAF3IP2* rs76228616 SNP: age, BMI, viral load, CD4 cells count, and two SNPs in *CYP2B6* gene and one SNP in *HCP5* gene that we had found significantly associated in our previous studies in the same population [11,12]. This analysis confirmed the significant involvement of *TRAF3IP2* (rs76228616) and *CYP2B6* (rs3745274 and rs28399499) in the susceptibility to SJS/TEN (Table 3). The final model explains about 24% of the susceptibility to SJS/TEN.

We also searched for possible interactions among *TRAF3IP2* SNP, the two *CYP2B6* SNPs and SJS/TEN susceptibility: Table 4 shows the three way-contingency table with the distribution of the rs76228616 and rs28399499 SNPs in SJS/TEN subjects and in controls. The log-linear analysis shows an interesting significant interaction among the three factors (*p* = 0.02). Considering in the log-linear analysis the other *CYP2B6* SNP (rs3745274), the interaction did not reach the statistical significance (*p* = 0.08, data not shown).

**Table 2:** *TRAF3IP2* genotypes and alleles distribution in Steven–Johnson Syndrome (SJS)/Toxic Epidermal Necrolysis (TEN) and controls.

SNP	SNP Location	Cases/Controls	N	Genotypes *			*p* ^§^	OR ^§^ (95% CI)	Alleles		*p*	OR (95% CI)
				wt	hz	var			Wildtype	Variant		
rs76228616 G>C	Exon 2	SJS/TEN	27	19	7	1	**0.027**	3.6 (1.2–10.95)	45	9	**0.012**	3.65 (1.33–10.01)
		Controls	77	69	8	0			146	8		
rs33980500 G>A	Exon 3	SJS/TEN	27	20	5	2	0.25	1.93 (0.67–5.54)	45	9	0.12	2.2 (0.88–5.49)
		Controls	78	66	11	1			143	13		
rs149246847 A>C		SJS/TEN	22	22	0	0	0.55	ND	44	0	0.55	ND
		Controls	50	47	3	0			97	3		
rs13190932 C>T		SJS/TEN	27	25	2	0	1	0.81 (0.16–4.17)	52	2	1	0.82 (0.16–4.07)
		Controls	78	71	7	0			149	7		
rs113085690 T>C	Intron 6	SJS/TEN	27	27	0	0	1	ND	30	0	1	ND
		Controls	50	49	1	0			55	1		
rs115622353 C>G	Exon 7	SJS/TEN	27	26	1	0	0.65	0.44 (0.05–4.17)	53	1	0.66	0.45 (0.05–4.16)
		Controls	50	46	4	0			96	4		
rs34659678 G>A		SJS/TEN	25	25	0	0	0.29	ND	50	0	0.3	ND
		Controls	50	46	4	0			96	4		
rs112619881 G>A		SJS/TEN	26	24	2	0	0.49	0.51 (0.1–2.66)	50	2	0.5	0.53 (0.11–2.7)
		Controls	50	43	7	0			93	7		
rs2280985 T>C	Intron 9	SJS/TEN	27	25	2	0	1	0.69 (0.12–3.8)	52	2	1	0.7 (0.13–3.74)
		Controls	48	43	5	0			91	5		
rs6912112 T>C		SJS/TEN	27	12	11	4	0.54	1.32 (0.54–3.17)	35	19	0.39	1.38 (0.72–2.67)
		Controls	78	40	32	6			112	44		
rs138485893 T>C	Exon 10	SJS/TEN	27	27	0	0	0.57	ND	54	0	0.57	ND
		Controls	78	75	3	0			153	3		

Significant associations are reported in bold; * Genotypes definition: wt = wildtype homozygote, hz = heterozygote, var = variant homozygote; **^§^** The *p* values and the ORs at genotypic level were calculated considering together the heterozygotes and variant homozygotes.

**Table 3:** Results of multiple logistic regression analysis.

Variables	B	*p*	OR (95% CI)	*R*^2^ (Cox and Snell)
age	0.065	0.142	1.07 (0.98–1.16)	0.246
Viral load (baseline)	0.00	0.311	1.00	
BMI	0.017	0.815	1.02 (0.88–1.17)	
CD4_50 (baseline)	0.111	0.083	1.12 (0.99–1.27)	
*TRAF3IP2* rs76228616	1.479	**0.027**	4.39 (1.19–16.23)	
*CYP2B6* rs3745274	1.147	**0.006**	3.15 (1.38–7.19)	
*CYP2B6* rs28399499	1.579	**0.011**	4.85 (1.43–16.4)	
*HCP5* rs3099844	0.595	0.158	1.81 (0.79–4.14)	

Significant associations are reported in bold.

**Table 4:** Interaction among rs28399499 (*CYP2B6*), rs76228616 (*TRAF3IP2*) and SJS/TEN: three way contingency table analysis by log-linear model.

		*CYP2B6* rs28399499			
		TT		TC + CC	
		SJS/TEN	Controls	SJS/TEN	Controls
*TRAF3IP2* rs76228616	GG	13	61	6	8
	GC + CC	7	8	1	0

XYZ interaction *p* = 0.02; X = *CYP2B6* rs28399499; Y = *TRAF3IP2* rs76228616; Z = cases/controls.

## 3. Discussion

SJS/TEN is one of the most serious adverse reactions to NVP treatment. It is known that the incidence of SJS/TEN is higher in patients with AIDS (1 affected person every 1000 subjects) [4]; this is probably due to the massive use of NVP, especially in developing countries. Although several studies have been conducted to identify genetic factors involved in the disease susceptibility, there are still few data that could help to identify individuals that are more suitable to develop this adverse reaction.

Moreover, the majority of studies have investigated the role of *HLA* alleles and particularly have analyzed cohort of patients presenting SJS/TEN induced by treatment with allopurinol and carbamazepine [7,8,9,10,22,23]. Very recently, few studies have analyzed the genetic factors involved in SJS/TEN induced by NVP treatment; Carr *et al.* described an association between the disease and the HLA-C*04:01 in Malawian population [10]. In our previous studies, we had identified a strong association between SNPs in *CYP2B6* gene [11], the cytochrome involved in NVP metabolism and we had confirmed an association with *HCP5* gene [12], already reported in Japanese and Caucasian patients treated with Carbamazepine and Allopurinol.

In the present paper, we have investigated the role of *TRAF3IP2* gene and its variants in the SJS/TEN susceptibility. We hypothesized that this gene, involved in immune response and in NF-κB activation, could also be implicated in the SJS/TEN susceptibility. The protein Act1 interacts with other TRAF proteins such as TRAF2 and TRAF5, which play multiple signaling roles in the IL-17R and TNF receptor signaling pathways.

By full sequencing of *TRAF3IP2* gene, we identified eight exonic and three intronic variants. One polymorphism in exon 2, rs76228616, resulted significantly associated with a higher risk (OR = 3.65 (95% CI = 1.33–10.01)) to develop SJS/TEN. This SNP has been described with a low frequency in Asian and European populations, but with a higher frequency in African population: in the Yoruba population, the minor allele frequency is equal to 18%, whereas in the Kenyan population the minor allele frequency is 10% (data from Hapmap and 1000 genomes databases). There are no data in other African populations. The frequency in the Mozambican population seems to be lower: 17% in cases and 5.2% in controls. So, apparently, the frequency shows a cline from north to South Africa. It should be interesting to evaluate if SJS/TEN is more frequent in patients treated with NVP in Yoruba or in Kenya populations than in Mozambique. This SNP is a non coding exon variant, located in 5'UTR, and therefore it should not interfere with the structure of the mature protein, but we could hypothesize a regulatory role.

Finally, the significant statistical interaction among *TRAF3IP2* rs76228616, *CYP2B6* rs28399499 and SJS/TEN susceptibility (3 way log-linear analysis) could also suggest a possible epistatic relationship between the two genes in determining SJS/TEN appearance. Of course, the nature of this interaction should be verified at functional level.

Admittedly, two limitations of our study are the relatively small number of samples with SJS/TEN and the fact that we could not perform a replication study; however, it should be underlined that fortunately the SJS/TEN is a rare adverse drug reaction (about 1 per 1000 individuals with AIDS) and therefore the recruitment of large samples of patients from the same population and treated with the same drug is not easy. For these reasons, even if our sample size is not great enough to reach definitive conclusions, a sample of 27 SJS patients can be considered satisfactory for a preliminary study.

In conclusion our data suggest a genetic influence of a polymorphism in *TRAF3IP2* gene on the susceptibility to SJS/TEN. Validation studies in independent populations and in larger cohorts as well as functional studies, will be necessary to evaluate the significance of this genetic variant on susceptibility to SJS/TEN and to its pathogenesis.

## 4. Experimental Section

### 4.1. Patients

Patients were recruited in three DREAM (Drug Resource Enhancement against AIDS and Malnutrition) Centers in Mozambique. All subjects were selected from patients accessing NVP-based combination treatment and all were females. The study was authorized by the Minister of Health of Mozambique. The sample of study consisted of 27 patients that developed SJS/TEN as adverse drug reaction and 78 matched controls that did not develop any adverse reaction after at least 4 year of NVP treatment. Criteria of SJS/TEN diagnosis were previously described [10] and clinical baseline characteristics of all patients are reported in Table 1. Baseline clinical characteristics (such as age, BMI, CD4 cells count, viral load and transaminases levels) did not present significant differences between cases and controls at the beginning of NVP treatment.

### 4.2. Genotyping and Statistical Analysis

All ten exons of *TRAF3IP2* gene, including the intron/exon boundaries, were amplified by PCR and analyzed by direct sequencing (ABI 3130xl Automated Sequencer (Applied Biosystems, Foster City, CA, USA)). Primers sequences and PCR conditions are reported in Table S1. The Hardy–Weinberg equilibrium was verified for all identified SNPs by the Pearson χ^2^ test. The same test has been used to evaluate differences in allelic, genotypic (1 degree of freedom (df), considering together the heterozygotes and variant homozygotes) and haplotypic frequencies between cases and controls. Haplotypes were inferred using Arlequin, version 3.5 [24]. Odds ratios (OR) with 95% CI were calculated. A binary logistic regression analysis was performed considering the occurrence of SJS/TEN as dependent variable and including as independent variables the *TRAF3IP2* rs76228616 (5'UTR) SNP, the *CYP2B6* rs28399499 (T983C, p.Ile328Thr) and rs3745274 (G516T, p.Gln172His)) SNPs, the rs3099844 (downstream *HCP5* gene) SNP, age, BMI and CD4 levels. Considering the result of multivariate analysis, a log-linear analysis (by a three way contingency table) was performed to test the possible interactions between the *TRAF3IP2* rs76228616 and *CYP2B6* rs28399499 SNPs in determining SJS/TEN and between the TRAF3IP2 rs76228616 and CYP2B6 rs3745274 SNPs. All statistical analyses were performed by the SPSS program ver. 19 (IBM Corp, Armonk, NY, USA).

## Supplementary Materials

Supplementary materials can be found at http://www.mdpi.com/1422-0067/16/03/5830/s1.
