# Supplementary material for: A Pharmacogenetics Study in Mozambican Patients Treated with Nevirapine: Full Resequencing of TRAF3IP2 Gene Shows a Novel Association with SJS/TEN Susceptibility
Source: Int J Mol Sci. 2015 Mar 12;16(3):5830–8. doi: 10.3390/ijms16035830 (PMC4394508; doi:10.3390/ijms16035830)
Supplement: Supplementary file 1 [file ijms-16-05830-s001.pdf]

Supplementary Information

Table S1. Primers sequences and PCR conditions.

Exons	Primers	Annealing	Amplified Fragment Length
**1**	**Fw**: 5'-CGATCTCCTGACCTCGTGAT-3'<br>**Rev**: 5'-GGGAGACGAACACAGAGGAA-3'	60 °C	700 bp
**2**	**Fw**: 5'-TCCCCTCTAGATGCATCACC-3'<br>**Rev**: 5'-GTTGGGAGCACTGACTGGTT-3'	58 °C	359 bp
**3**	**Fw**: 5'-AGGGCTTTGCAAGCACATTG-3'<br>**Rev**: 5'-CATTCTGACCTGTTCATGCCA-3'	58 °C	1079 bp
**4**	**Fw**: 5'-TACGGCAAACATCACAGAGG-3'<br>**Rev**: 5'-TGCATTGGCATAATTCCTTTC-3'	58 °C	399 bp
**5**	**Fw**: 5'-TTCTGTCCCTGAAGGTTTGC-3'<br>**Rev**: 5'-CTTGCAGTGAGCTGAGATCG-3'	61 °C	362 bp
**6**	**Fw**: 5'-AGGACAGCATACAACGTAGGC-3'<br>**Rev**: 5'-TATGTGGTCAGCATTAGCACT-3'	60 °C	514 bp
**7**	**Fw**: 5'-GCTCTGCATCTTGAGAGAACC-3'<br>**Rev**: 5'-ACTCGACTGCCCTGTCTTCT-3'	62 °C	810 bp
**8**	**Fw**: 5'-TTTGCTCTGCATGTTCAAGG-3'<br>**Rev**: 5'-CTGGTCTCTGGGGAAGAATG-3'	62 °C	399 bp
**9**	**Fw**: 5'-CATGTGGACGTGCATGATTA-3'<br>**Rev**: 5'-CACAGCGAGACTCCATCTCA-3'	60 °C	297 bp
**10**	**Fw**: 5'-ATGCTGTGCACATGTGGTTT-3'<br>**Rev**: 5'-TTGTTGATGTCCCTGGAAGC-3'	60 °C	916 bp
